# Effects of taurine on resting-state fMRI activity in spontaneously hypertensive rats

**DOI:** 10.1371/journal.pone.0181122

**Published:** 2017-07-10

**Authors:** Vincent Chin-Hung Chen, Tsai-Ching Hsu, Li-Jeng Chen, Hong-Chun Chou, Jun-Cheng Weng, Bor-Show Tzang

**Affiliations:** 1 Department of Psychiatry, Chang Gung University, Taoyuan, Taiwan; 2 Department of Psychiatry, Chiayi Chang Gung Memorial Hospital, Chiayi, Taiwan; 3 Institute of Biochemistry, Microbiology and Immunology, Chung Shan Medical University, Taichung, Taiwan; 4 Clinical Laboratory, Chung Shan Medical University Hospital, Taichung, Taiwan; 5 Department of Medical Imaging and Radiological Sciences, Chung Shan Medical University, Taichung, Taiwan; 6 Department of Medical Imaging and Radiological Sciences, Chang Gung University, Taoyuan, Taiwan; 7 Department of Biochemistry, School of Medicine, Chung Shan Medical University, Taichung, Taiwan; Institute of Biochemistry and Biotechnology, TAIWAN

## Abstract

Attention deficit hyperactivity disorder (ADHD) is a global behavior illness among children and adults. To investigate the effects of taurine on resting-state fMRI activity in ADHD, a spontaneously hypertensive rat (SHR) animal model was adopted. Significantly decreased serum C-reactive protein (CRP) was detected in rats of Wistar Kyoto (WKY) high-taurine group and significantly decreased interleukin (IL)-1β and CRP were detected in rats of SHR low-taurine and high-taurine groups. Moreover, significantly higher horizontal locomotion was detected in rats of WKY low-taurine and SHR low-taurine groups than in those of controls. In contrast, significantly lower horizontal locomotion was detected in rats of the SHR high-taurine group than in those of the SHR control group. Additionally, significantly lower functional connectivity (FC) and mean amplitude of low-frequency fluctuation (mALFF) in the bilateral hippocampus in rats of WKY high-taurine and SHR high-taurine groups was detected. Notably, the mALFF in rats of the SHR low-taurine and high-taurine groups was significantly lower than in those of the SHR control group. These findings suggest that the administration of a high-dose taurine probably improves hyperactive behavior in SHR rats by ameliorating the inflammatory cytokines and modulating brain functional signals in SHR rats.

## Introduction

Attention deficit hyperactivity disorder (ADHD) is a very common developmental disorder in both children and adults worldwide, with prevalences of 5–10% [1] and 3–5% [2], respectively. Although the exact etiology of ADHD is still unclear, various causes of the pathogenesis of ADHD have been suggested. Inflammation has been associated with various neuropsychiatric illnesses, including ADHD. Elevated pro-inflammatory cytokines such as interleukin (IL)-1, IL-6, CRP and tumor necrosis factor alpha (TNF-α), are known as common pathogenic parts of schizophrenia, ADHD and autism [3]. Children with extremely prematurely birth, recurrent, or persistently inflammation during the first 14 postnatal days are strongly associated with attention deficiency [4]. Accordingly, a systematic review article regarding inflammation among young people with neuropsychiatric diseases reveals that elevated inflammatory markers play critical roles in the development of ADHD [5].

Magnetic resonance imaging (MRI) has been utilized to evaluate the anatomic differences in brain areas between ADHD children and their controls [6–7]. Overall reductions in total brain volume have been reported in ADHD children than those of age- and gender-matched controls [8]. Indeed, evidences consistently show that the average brain volume in ADHD children is significantly smaller than in healthy controls [9]. Brain areas of particular interest in this regard are the prefrontal regions, basal ganglia, corpus callosum, and cerebellum. For instance, differences in distributions of grey and white matter in children with ADHD and healthy controls have been reported [10]. Since specific executive function impairments in particular brain areas of ADHD patients have been associated with the impaired behavioral phenomena [11], functional MRI (fMRI) was employed herein has been used as the method of choice to provide superior spatial resolution in ADHD cases. Based on fMRI measurement, the pathogenesis of ADHD is likely to involve a reduction in volume or function in specific brain areas, resulting in various behavioral problems such as abnormal cognitive processing, attention, motor planning, and speed of processing responses [12].

Taurine, which is known to be the richest amino acid in the central nervous system, performs various functions in the body, including antioxidant, anti-inflammation, osmoregulation, neuromodulation, membrane stabilization, embryogenesis and immune regulation [13–15]. Increasing wariness has recently been paid to the role of taurine in neuro-regulation. Taurine is an endogenous ligand for the glycine receptor in the nucleus accumbens, elevating the dopamine level in this area [16]. Additionally, taurine is reported to induce anti-anxiety effects in an animal model probably owing to its association with GABA and has been used as a natural anxiolytic, or anti-anxiety compound, although some studies reveal mixed results [17–19]. These findings strongly associate taurine with various ADHD-related neurotransmitters. Therefore, we intend to study the effects of taurine on serum inflammatory factors, horizontal locomotion and brain functional activity in an ADHD-like animal model.

## Materials and methods

### Animals and diets

As a valid and accepted animal model for ADHD, the spontaneously hypertensive rat (SHR) and its control, the Wistar-Kyoto (WKY) rat, were used in this study [20–21]. Three week-old male SHR/NCrlCrlj and WKY/NCrlCrlj rats (with a body weight of approximately 180g) were purchased from the National Laboratory Animal Center, Taipei, Taiwan. Rats of each the two species were separated into control (Control), low taurine (Low Tau) and high taurine (High Tau) groups (eight rats /group) and housed in an animal room at 22±2°C with a 12/12 h light-dark cycle. All protocols were followed under the supervision of the Institutional Animal Care and Use Committee at Chung Shan Medical University (IACUC approval number: 1671), following the Guide for the Care and Use of Laboratory Animals that was published by the United States National Institutes of Health. The dosage of taurine used in this study is according to a previous study [19]. All animals were provided with chow diets (Laboratory Rodent Diet 5001, PMI Nutrition International/Purina Mills LLC., USA) and water for one week for acclimation. Subsequently, the rats in the Control, Low Tau and High Tau groups were fed a chow diet, a low taurine diet (22.5 mmol/kg taurine), and a high taurine diet (45 mmol/kg taurine) for four weeks, respectively. Before the animals were sacrificed, horizontal locomotion and rs-fMIR was performed. Subsequently, the animals were sacrificed at an age of eight weeks by CO_2_ asphyxiation. Blood was obtained from the heart and stored at -80°C before analysis.

### Measurement of horizontal activity in an open-field assay

Horizontal activity was detected as described elsewhere [22]. All rats were set in an open-field device (acrylic cylinder; 40 cm diameter × 40 cm height) for two consecutive days prior to the test day to diminish any influences of the treating process. Briefly, each *rat* was put in the middle of the apparatus under ambient lighting, and then allowed to explore the open field for 90 minutes in the first two days. On day three, the rat was placed in the same open-field device. Horizontal activity was measured for 90 minutes as the total number of interruptions of the beam of a horizontal sensor (SCANET MV-10; Melquest Co., Ltd, Toyama, Japan). All behavioral evaluations were performed between 08:00 and 15:00 h.

### ELISA estimations for IL-1β and CRP

Serum CRP and IL-1β concentrations were measured in triplicate with a commercially available enzyme-linked immunosorbent assay kit (BD Pharmingen, San Diego, CA, USA) according to the instruction of manufacturer.

### Functional MRI acquisition

Whole brain images were acquired from all subgroups mentioned above and scanned by 7T MRI (Bruker BioSpin, Ettlingen, Germany). In preparation, each rat was anesthetized with 3.5% isoflurance mixed with 800 ml/min air. Before MRI experiments a short-acting tranquilizer and synthetic medicine, Domitor (0.1 ml/500 g), was injected. The sedation effect of Domitor was about half hour to one hour. It is commonly used in animal surgery and fMRI study to avoid BOLD signal contamination by isoflurance. During experiments, the temperature was maintained at around 37°C using hot pad. All functional images were acquired by gradient echo based echo planar imaging (EPI) with the following parameters: repetition time/echo time (TR/TE) = 2000ms /20 ms, resolution (voxel size) = 3.12 x 3.12 x 1 mm^3^, slice number = 12, number of repetition = 300, and the scan time = 10 min.

### Functional image pre-processing

The raw data were first converted into ANALYZE format. The preprocess was then performed, including slice timing, realignment, denoise, detrending and filtering, using Statistical Parametric Mapping (SPM8, Wellcome Department of Cognitive Neurology, London, UK) and Resting-State fMRI Data Analysis Toolkit (REST1.8, Lab of Cognitive Neuroscience and Learning, Beijing Normal University, China) [23]. For motion correction, all EPI images in each run were realigned to first image. To reduce the noise, all data were spatially smoothed by a Gaussian kernel with full width at half maximum (FWHM) of [0.6 mm, 0.6 mm, 2 mm]. To cut down the influences of low-frequency linear and quadratic drifts and physiological signals, detrending and band-pass temporal filtering (0.01–0.12 Hz) were adopted on the time series of each voxel.

### Functional connectivity

Functional connectivity analysis is a seed-based analysis that can observe the connectivity between brain regions that share functional properties. It indicates selecting regions of interest (ROIs) and correlating the average blood oxygenation level dependent (BOLD) time course of voxels within these ROIs with each other as well as with the time courses of all other voxels. Using the rat brain in stereotaxic coordinates as a reference, seed region was prescribed for each choosing brain area center, then extract a spherical region with a 0.5mm radius. Spherical seeds were prescribed for each network. We used the posterior cingulate cortex (PCC) as the seed region to identify the default mode network (DMN) [24]. In addition, we used the bilateral amygdala, hippocampus, thalamus and motor as seed points to construct the functional connectivity map. To assess bilateral connectivity, we calculated the correlation between seed point and other sides. A circular ROI was selected on the contralateral side of the seed regions, with the shortest axis of the brain area as the radius; the ROI size in each group was controlled to be the same. We used Functional Magnetic Resonance Imaging of the Brain Software Library (FMRIB Software Library, FSL, Oxford, UK) with FSL-View to make the 3D mask for calculating the correlation between seed points and other sides, and the mean signals in the functional connectivity map. Finally, the average z-scores of the two groups were determined.

### Amplitude of low frequency fluctuations

The amplitude of low frequency fluctuations (ALFF) calculates low-frequencies spectral power (0.01–0.12 Hz) of BOLD signal voxel-by-voxel [25]. The ALFF measures the correlation of low frequency fluctuations of BOLD signal at each brain region, which is used to proxy the absolute intensity of spontaneous brain activity [26]. The ALFF have numerous similarities with fluctuations in neural metabolic, hemodynamic, and neurophysiological parameters [27]. Therefore, the ALFF during resting state is considered to be physiologically meaningful and reflective of spontaneous neural activity [28]. For calculating mALFF, the time series was first converted to the frequency domain using a Fast Fourier Transform for a given voxel. The square root of the power spectrum was measured, averaged and normalized across a predefined frequency interval, which was termed the ALFF at the given voxel [28]. To reduce the global effects of variability across rats, the ALFF of each voxel was divided by the global mean ALFF value for each subject, resulting in a relative ALFF, mALFF. We then performed two-sample t-tests with FDR correction to assess the difference in mALFF between the SHR and WKY rats.

### Statistical analysis

All data are presented as mean ± standard error. Two-way analysis of variance (ANOVA) followed by Dunnett's test was performed to evaluate the horizontal activity data. The comparisons among groups were performed using GraphPad Prism 5 software (GraphPad Software, Inc., La Jolla, CA, USA) by one-way analysis of variance (One-way ANOVA) followed by Tukey multiple comparisons test. A value of P<0.05 was considered statistically significant. Additionally, to investigate the associations between fMRI indices (FC and mALFF) and behavior/biochemical measures (horizontal activity, IL-1β and CRP), the scatter plots were performed.

## Results

### Effects of taurine on horizontal locomotion in WKY and SHR rats

**Fig 1:**
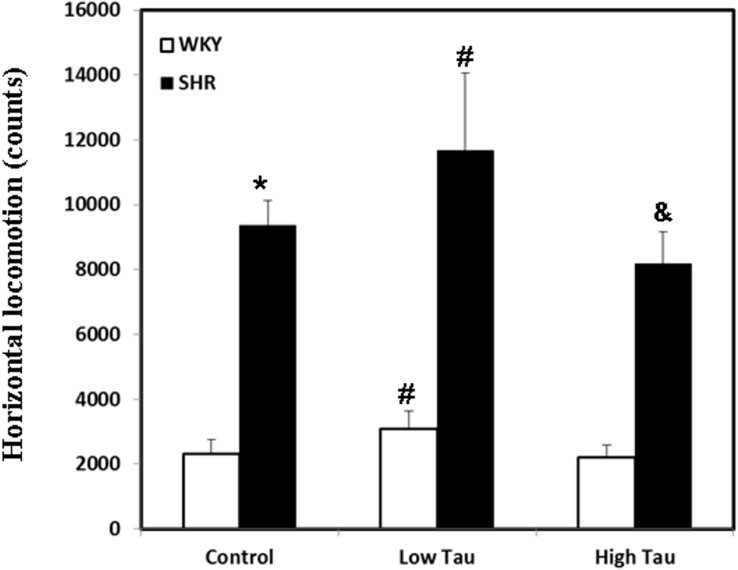
Horizontal locomotion of WKY and SHR rats. Both WKY and SHR rats were divided into three subgroups, including control group (Control), low dose taurine group (Low Tau), and high dose taurine group (High Tau). The data are expressed as the mean ± standard error. The symbols, * (WKY Control vs. SHR Control), # (WKY Low Tau vs. WKY Control; SHR Low Tau vs. SHR Control), & (SHR High Tau vs. SHR Control) indicate statistically significant (P<0.05).

The effect of taurine on locomotion of SHR and WKY rats was examined by detecting their horizontal locomotion. Rats of the SHR control group exhibited significantly higher horizontal locomotion than those of the WKY control group (Fig 1). Significantly higher horizontal locomotion was detected in rats of WKY low-taurine than in those of WKY control group (Fig 1). Similar result was observed that SHR rats that were fed with low-dose taurine exhibited significantly higher horizontal locomotion than in those of the SHR control group (Fig 1). Conversely, the SHR rats that were fed with a high dose of taurine exhibited significantly less horizontal locomotion than in those of the SHR control group (Fig 1).

### Effects of taurine on serum IL-1β and CRP levels in WKY and SHR rats

**Fig 2:**
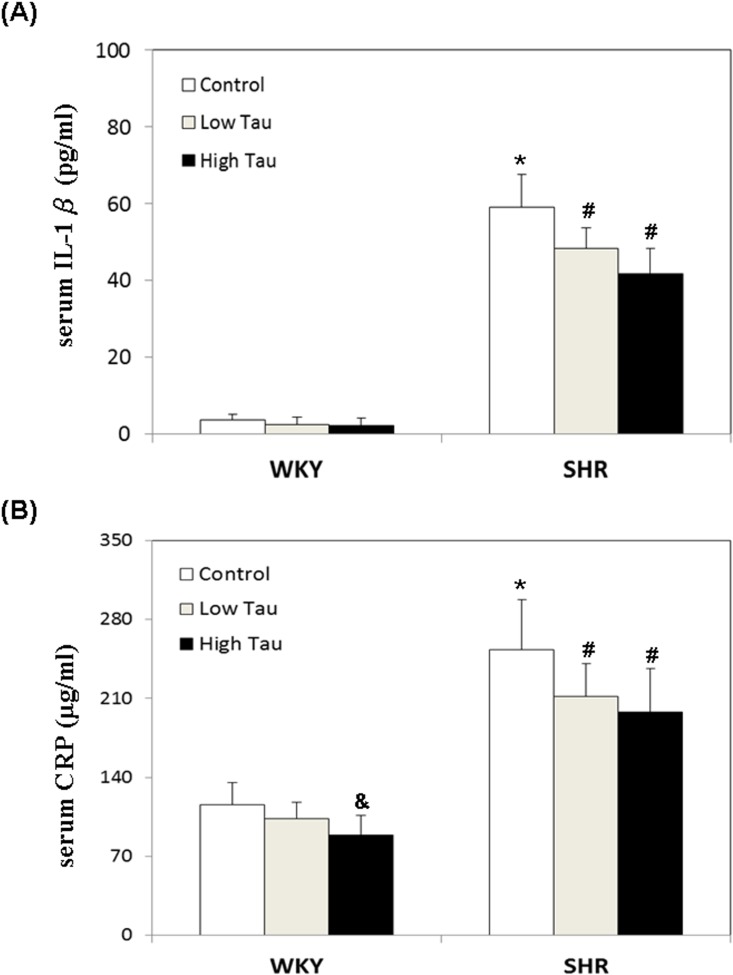
**Serum (A) IL-1**β **and (B) CRP levels in WKY and SHR rats**. Both WKY and SHR rats were divided into three subgroups, including control group (Control), low dose taurine group (Low Tau), and high dose taurine group (High Tau). The data are expressed as the mean ± standard error. The symbols, * (WKY Control vs. SHR Control), # (SHR Low Tau or SHR High Tau vs. SHR Control) and & (WKY High Tau vs. WKY Control), indicate statistically significant (P<0.05).

Elevation of inflammatory factors induces the development of neurological and mental pathologies, including ADHD. This study further investigates the effects of taurine on inflammatory factors, including IL-1β and CRP, in both SHR and WKY rats. Significantly higher level of serum IL-1β was detected in rats of the SHR control group than in those of the WKY control group (Fig 2A). No significant difference between serum IL-1β levels was detected among WKY rats that were fed with low or high dose of taurine and those of the control group (Fig 2A). A significantly lower serum IL-1β levels were detected in SHR rats that were fed with low or high dose of taurine that in those of the SHR control group (Fig 2A). Significantly higher level of serum CRP was detected in rats of the SHR control group than in those of the WKY control group (Fig 2B). Significantly lower serum CRP level was detected in rats of the WKY high-taurine group than in those of the WKY control group (Fig 2B). Notably, the serum CRP level in rats of SHR low and high taurine groups were significantly lower than in those of the SHR control group (Fig 2B).

### Effects of taurine on functional connectivity

**Fig 3:**
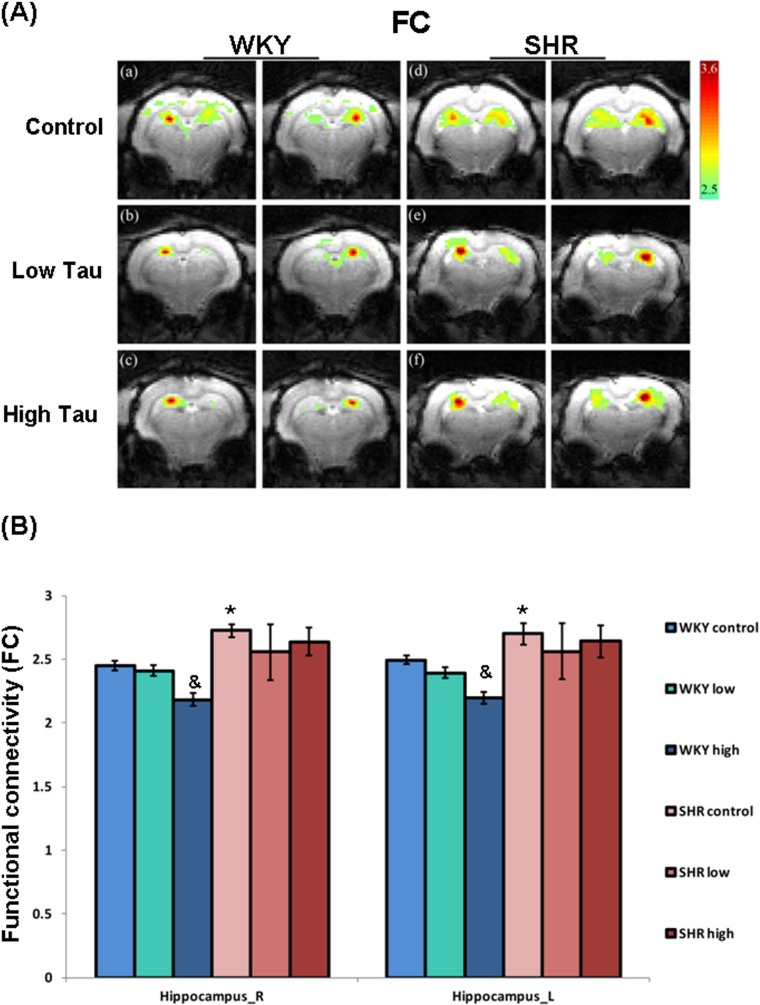
The functional connectivity of bilateral hippocampus in WKY and SHR rats. The (A) representative images of functional connectivity (FC) in bilateral hippocampus of (a-c) WKY and (d-f) SHR rats. Both WKY and SHR rats were divided into three subgroups, including (a, d) control group (Control), (b, e) low dose taurine group (Low Tau), and (c, f) high dose taurine group (High Tau). The color bar represents z-scores. (B) The FC of bilateral hippocampus in WKY and SHR rats. The data are expressed as the mean ± standard error. The symbols, * (WKY Control vs. SHR Control) and & (WKY High Tau vs. WKY Control), indicate statistically significant (P<0.05).

To investigate the effects of taurine on functional signals in various brain regions of SHR and WKY rats, the signal of functional connectivity (FC) was detected using resting-state functional magnetic resonance imaging (rs-fMRI). The FC maps are presented as individual animals, which were composed of six individual representative rat brain maps, respectively. The color bar represents z-scores. In Fig 3, the FC of the bilateral hippocampus in rats of the SHR control group was significantly higher than in those of WKY control group. The FC of the bilateral hippocampus in rats of the WKY high-taurine group was significantly lower than in those of the WKY control group (Fig 3B). However, no significant variation of the FC in the bilateral hippocampus was detected in rats of the SHR low-taurine and high-taurine groups than in those of the SHR control group (Fig 3B). No significant variation in the FC of the other brain regions was detected among the groups (data not shown).

### Effects of taurine on mean amplitude of low-frequency fluctuations

**Fig 4:**
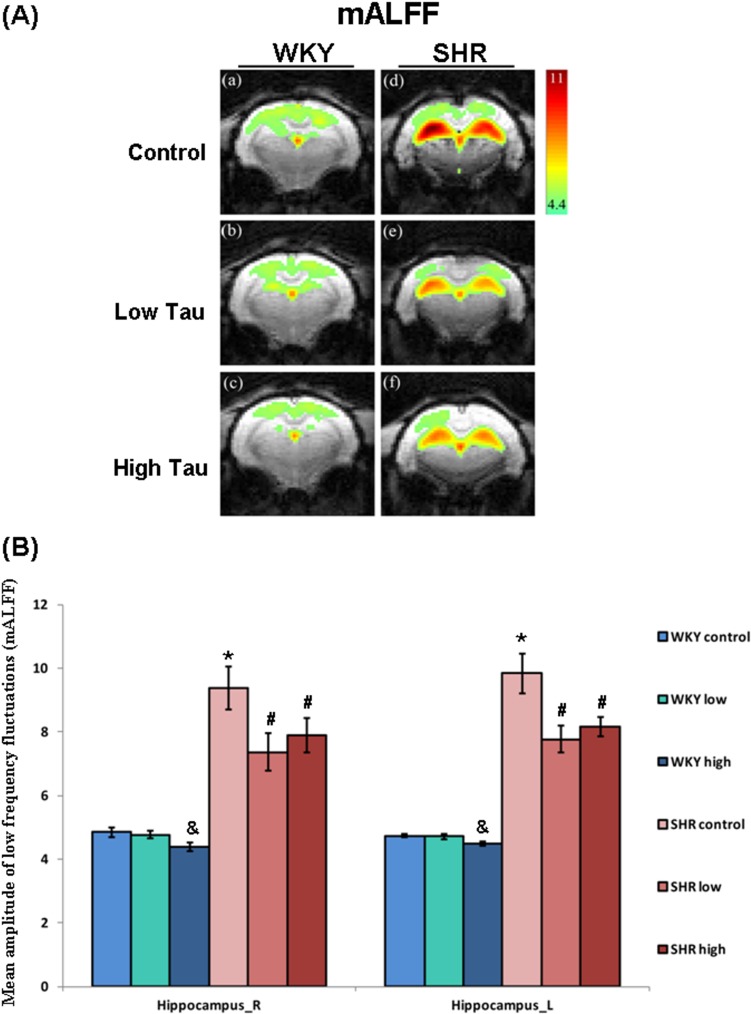
The mALFF of bilateral hippocampus in WKY and SHR rats. The (A) representative images of mALFF in hippocampus of (a-c) WKY and (d-f) SHR rats. Both WKY and SHR rats were divided into three subgroups, including (a, d) control group (Control), (b, e) low dose taurine group (Low Tau), and (c, f) high dose taurine group (High Tau). The color bar represents z-scores. (B) The mALFF of bilateral hippocampus in WKY and SHR rats. The data are expressed as the mean ± standard error. The symbols, * (WKY Control vs. SHR Control), # (SHR Low Tau or SHR High Tau vs. SHR Control) and & (WKY High Tau vs. WKY Control), indicate statistically significant (P<0.05).

The signal of mean amplitude of low-frequency fluctuation (mALFF) was detected to clarify the effects of taurine in various regions of rat brain. The mALFF maps are presented as individual animals, which were composed of six individual representative rat brain maps, respectively. The color bar represents z-scores. In Fig 4, the mALFF of the bilateral hippocampus in rats of the SHR control group was significantly higher than that in rats of the WKY control group. The mALFF of the bilateral hippocampus in rats of the WKY high-taurine group was significantly lower than in those of the WKY control group (Fig 4B). After treatment with a low or high dose of taurine, the mALFF of the bilateral hippocampus in rats of the SHR low-taurine and high-taurine groups was significantly lower than in those of the SHR control group (Fig 4B). No significant variation in mALFF in the other brain regions was detected among groups (data not shown).

### Correlative analysis of fMRI indices biochemical measures

**Fig 5:**
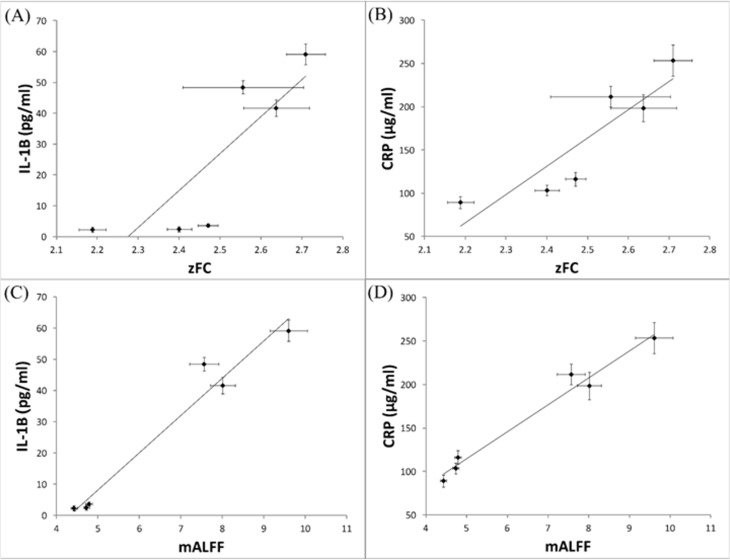
The correlation between fMRI indices and inflammatory-cytokine levels in SHR rats. The scatter plots were performed to evaluate the correlation between (A) FC and IL-1β (r = 0.85), (B) FC and CRP (r = 0.90), (C) mALFF and IL-1β (r = 0.98), and (D) mALFF and CRP (r = 0.99).

To investigate the correlation between fMRI indices, including FC and mALFF, and biochemical measures, including horizontal activity, IL-1β and CRP, the scatter plots were performed. Accordingly, positive correlation between FC and IL-1β (r = 0.85), FC and CRP (r = 0.90), mALFF and IL-1β (r = 0.98), and mALFF and CRP (r = 0.99) were detected (Fig 5). However, no significant correlation was found between FC and horizontal activity or mALFF and horizontal activity (data not shown).

## Discussion

Accumulating evidence suggests a strongly pathophysiological relationship between inflammation and attention-deficit hyperactivity disorder (ADHD) [5]. Elevated levels of pro-inflammatory cytokines such as interleukin (IL)-1 and C-reactive protein (CRP) are known to be diagnostic markers and crucial pathogenic factor of ADHD [3, 29–30]. When cell damage occurs, CRP binds to phosphocholine on cell membranes and promotes the binding of complements [31]. Evidence reveals that IL-1 has both neuromodulatory and neurodevelopmental functions that involve turnover neurotransmitters in various brain regions [32]. Interleukin-1 (IL-1α and IL-1β), IL-1Ra, and IL-1 receptors are found to be expressed in the brain [33]. Especially in relation to ADHD, IL-1β induces changes in dopamine (DA) and norepinephrine (NE) in the prefrontal cortex. Various studies have also shown that the systemic administration of IL-1β enhances DA and NE utilization in the prefrontal cortex (PFC) in both mice and rats [32, 34]. In this work, serum CRP and IL-1β levels were significantly lower in SHR rats that were administered a low dose or high dose of taurine. These results suggest that taurine can ameliorate inflammatory factors, resulting in ameliorating ADHD-like behaviors, such as hyperactivity, in SHR rats.

Functional magnetic resonance imaging (fMRI) is a specialized MRI method that is used to measure hemodynamic responses; it provides images of the brain microvasculature in which the image contrast reflects blood oxygen level [35]. The signals of blood-oxygen-level-dependent (BOLD) dominate the mechanism that gives rise to functional connectivity in the resting human brain [36]. Low-frequency temporal components (<0.1Hz) in resting-state BOLD fMRI reflect spontaneous fluctuations in brain physiology and metabolism, which regards as imaging markers of brain function [37]. Several indices of spontaneous low-frequency fluctuations of BOLD fMRI, including the amplitude of low-frequency fluctuations (ALFF) [38], regional homogeneity (ReHo) and functional connectivity (FC) [39], have been introduced to evaluate resting brain function in patients with various psychiatric disorders, such as ADHD [40]. Evidence suggests that increased hippocampus volume may reflect neural activities such as temporal processing and delay aversion within the hippocampus in ADHD [8]. However, these findings are not very stable among samples from different ADHD patients and the contradictory findings may be related to the different locations of alterations in the complex circuits that are responsible for the various symptoms of ADHD [41–42]. A previous study reported that ADHD patients exhibited higher ALFF values in the left superior frontal gyrus and sensorimotor cortex (SMC) and suggested irregular frontal activities in the resting state that are associated with the underlying physiopathology of ADHD [43]. Other evidence indicates that ADHD patients exhibit impaired executive function and abnormal fMRI indices, such as increased ALFF in both left and right globus pallidus and right dorsal superior frontal gyrus, and increased FC in the frontostriatal circuit, relative to healthy controls [44]. Notably, the SHR rats that were fed with taurine exhibited significantly lower mALFF values than those in SHR control group in the hippocampus, as well as less horizontal locomotion and lower levels of inflammatory proteins. These findings suggest that taurine mitigates mALFF in SHR rats and probably result in reduced ADHD-like behavior in SHR rats. However, further investigations are required to elucidate the precise associations between fMRI indices and ADHD symptoms.

An interesting result was observed in this study. The horizontal locomotion of SHR rats that were fed with a low dose of taurine was significantly higher than that of rats of the SHR control group. However, the SHR rats that were fed with a high dose of taurine exhibited significantly less horizontal locomotion, owing to the effect of the dose of taurine. Accordingly, a recent study found that glycine receptors were activated by administrating a low-dose taurine (0.5mM), whereas a higher dose of taurine (3mM) induced both glycine and gamma-aminobutyric acid A (GABAA) receptors in preoptic hypothalamic area (PHA) neurons [45]. Another study reported that taurine supplementation increases locomotor activity and anxiety whereas taurine injection suppressed locomotor activity and anxiety [18]. It indicates that taurine could induce opposite effects through different ways of adminstration. However, investigations are needed to clarify whether high-dose taurine supplemation is similar to the effect of lower-dosage taurine injection. Although further works are required to elucidate the influences of different dosages of taurine as well as its administrative methods, these findings may provide a possibie explanation of why taurine has ameloriate effects on ADHD-like behaviors in SHR rats.

Although the cause of ADHD remains unclear, impaired prefrontal cortex (PFC) circuits plays crucial roles in the pathogenesis of ADHD [46]. Pharmacotherapy to treat ADHD is based on evidence that norepinephrine and dopamine are extensively involved in various functional brain regions such as motor control, working memory, attention, and executive function [47]. Therefore, a reduced GABA concentration was detected in cases of ADHD and associated with its pathogenesis [48]. Taurine is the second most abundant neurotransmitter in the central nervous system, which plays important roles in brain neuronal proliferation, stem cell proliferation, and differentiation [49]. Clinically, taurine can be directly used in the treatment of brain development because it has no toxic effects on humans [49]. Also, taurine can interact with dopamine in the striatum [50] and induces the activation of extrasynaptic GABA A receptors in the thalamus [51], taurine can elevate dopamine levels in the nucleus accumbens [16, 52]. The above findings suggest diverse roles of taurine in modulating ADHD-related neurotransmitters. Although further investigations are needed to confirm the precise effect of taurine on neurotransmitters in cases of ADHD, and the multiple modulatory effects of taurine, especially in activating the GABA A receptor and elevating dopamine levels, this study may provide a rational explanation of taurine’s amelioration of ADHD-like symptoms in SHR rats by modulating neurotransmitters, such as GABA, dopamine and glutamate or their receptors, in the brain.

## Conclusion

This study is the first to reveals the beneficial effects of taurine on an ADHD animal model. The treatment of SHR rats with a high dose of taurine significantly reduces the levels of pro-inflammatory cytokines and horizontal locomotor activity. Treatment with a low or high dose of taurine caused the FC of the bilateral hippocampus in SHR rats to return to the levels in rats of the WKY control group. Taurine significantly reduces the mALFF of bilateral hippocampus in SHR rats. Although no significant correlation was found between FC and horizontal activity or mALFF and horizontal activity, this study still disclosers the ameliorative effect of high-dose taurine on hyperactivity in SHR rats and suggests that taurine may be an alternative treatment for ADHD. However, further animal and clinical studies are required to determine the efficacy, safety, optimal dosage and precise mechanism of taurine for clinical purposes.
