# Correction: Effects of taurine on resting-state fMRI activity in spontaneously hypertensive rats

**DOI:** 10.1371/journal.pone.0190203

**Published:** 2017-12-19

**Authors:** Vincent Chin-Hung Chen, Tsai-Ching Hsu, Li-Jeng Chen, Hong-Chun Chou, Jun-Cheng Weng, Bor-Show Tzang

In the fourth sentence in the “Animals and diets” section within the Materials and methods, the IACUC approval number is listed incorrectly. The correct sentence is: All protocols were followed under the supervision of the Institutional Animal Care and Use Committee at Chung Shan Medical University (IACUC approval number: 1583), following the Guide for the Care and Use of Laboratory Animals that was published by the United States National Institutes of Health.

## References

[1] Chen VC-H, Hsu T-C, Chen L-J, Chou H-C, Weng J-C, Tzang B-S (2017) Effects of taurine on resting-state fMRI activity in spontaneously hypertensive rats. PLoS ONE 12(7): e0181122 https://doi.org/10.1371/journal.pone.0181122 28700674 10.1371/journal.pone.0181122 PMC5507323

